# Extracts and Gleanings

**Published:** 1875-08

**Authors:** 


### Extracts and Gleanings.

*The Curative Treatment of Insanity by Chlorhydrate of Morphia.* Dr. Voisin, of the Salpétriére Hospital, (*Bulletin General des Thérapeutique*, March, 1874,) arrives at the following conclusions:

If the malady be recent and not complicated by successions of delusions, the remedy keeps it from progressing, and stops the development of secondary or tertiary stages. Moreover, it calms the general or partial agitation in a time varying from two to three hours after a sufficient quantity has been injected. This dose is very variable, and is only arrived at by experience. The manner in which the delirium disappears is interesting. The delusions, hallucinations, etc., seem to separate off from one another and no longer to form part of the same whole; they disappear one after another, and the patient replies to questions and recognizes that he has been ill, his memory returns, and he recounts a series of facts relating to his entry to the hospital, he writes to his family and allows himself to be treated medically without offering any resistance. It seems as if the symptoms of insanity disappear inversely in the order of their occurrence. Thus the patients who have begun by hallucinations, followed by delusions, in their recovery lose the latter before the former, and though the hallucinations may remain for some days they do not believe in them. The indications for the remedy are: melancholia, with or without hallucinations; ecstacy; suicidal ideas; religious delusions; maniacal excitement; the various forms of neuralgia which are so common among the insane, and especially in the women, which often determine the especial character of the delusions, giving, *e. g.*, the notion that they are being electrified. The peculiar form of *folie circulaire*, which is generally thought to be incurable, has been very successfully treated by it.

The contra-indications for its use are: inflammatory symptoms, epileptic insanity, and general paralysis. In that form resulting from atheroma of the arteries, it may cause mischief from congestion, leading thence to hemorrhage.

Of the twenty-five cases quoted which were cured, six were affected with general mania with hallucinations and incoherence. The strongest dose employed in these six cases were twenty-one centigrammes daily, and the smallest was thirty-one milligrammes da the average duration of the treatment being four months. The other successful cases were of melancholic form, with more or less suicidal and homicidal complications.

Of the five unsuccessful cases, four had delusions of wealth and power, and one had certain febrile complications, a condition which has been shown by later experience to be totally opposed to the proper use of the remedy.—*New Remedies.*

*Bromide of Potassium in Strychnine Poisoning.*—SIR: Thinking the following might be of some interest to the profession, I take this opportunity of presenting it.

Mr. T., a farmer 25 years of age, of temperate habits, while suffering from mental aberration, procured on the 18th inst., twelve grains of strychnia, representing to the apothecary that he wished to poison some rats. Returning home, he retired as usual. About 5 o'clock on the next morning he arose, mixed half the quantity of strychnia in a cup of water, and drank it. His mother hearing him, and mistrusting from some unusual action that all was not right, hastily arose and soon drew from him the confession that he had swallowed "poison" with suicidal intent. I was summoned immediately, first seeing him one-half hour after he had swallowed the potion. I found him in bed, lying upon his back, unable to move his arms or legs, his head drawn back and jaws quite stiff; yet his intellect, so far as I could discover, was perfectly clear. He answered my questions in a manner which led me to suppose he understood them, and said he had taken strychnine. I put my hand upon his head, and immediately there occurred a spasm, resembling the shock one exhibits from a sudden and intense discharge from an electric battery. These shocks or spasms could be produced at will as often as any one touched him, and were repeated from every two to four minutes, whether he was disturbed or not.

Mixing a desertspoonful of powdered mustard in some water, and adding a drachm of Squibb's fluid extract of ipecac, I succeeded, after some perseverance, in the midst of successive spasms, in getting him to swallow all of it. Emesis occurred in about ten minutes, the spasms being full as free as the emesis. He drank nearly a quart of warm water. This was also freely vomited. After the vomiting, the spasms still continuing, I gave twenty grains of bromide of potash. He immediately exhibited the most profuse perspiration I ever beheld. I thought him dying. In fifteen minutes more I exhibited another fifteen grains of the bromide This I repeated every fifteen minutes till one hundred grains had been taken, and the spasms had gradually subsided and now entirely ceased. I ordered the bromide each two hours after, and left him feeling quite comfortable.

On seeing him the next day, he complained of nothing but soreness of the muscles generally. Said he felt as though he had been skating on the ice. He recovered from this slowly, and now is apparently as well as before the drug was taken.

My reason for using the bromide instead of the remedies usually recommended was, that it seemed to be the only medicine I had at hand indicated rationally from his condition, having neglected the precaution to take chloroform or ether with me, or other things used to control spasms or to act as an antidote.

From the result of this case, I conclude that bromide of potassium may prove to be a serviceable remedy in such cases.—*Medical Record.*

*Phosphide of Zinc.*—At a recent meeting of the Obstetrical Society (*Lancet*, Feb. 27,) Mr. J. Ashburton Thompson communicated a short paper exemplifying the advantage of employing phosphide of zinc in the treatment of chlorosis and anæmia. It succeeded in relieving the symptoms where iron had failed, and that rapidly. Phosphorous was of great value in the treatment of patients recovering from uterine hemorrhage, and in all cases of anæmia, and seemed to exercise a specific influence upon the neuralgia so often met with in these cases, encouraging the general nutrition of the body. Free phosphorus is not the treacherous poison it has hitherto been considered to be. It is a fatal and potent poison, it is true, but its therapeutic effects may be obtained with precision and perfect safety. If proper formula be employed, no apprehension of unexpected or uncontrollable poisonous effects of a therapeutic dose need hinder its general employment.—*Phil. Med. Times.*

*On Hydrocele of the Neck—A Clinical Note.*—In March last year, Mrs. D——, from Wolverhampton, called on me with her youngest child, a healthy looking boy two years old, who had a tumor on the left side of the neck. The growth was noticed very soon after birth, and had very steadily increased to its present size. When the clothes were removed, I found a round smooth mass occupying the whole left side of the neck, and projecting over the clavicle on to the upper part of the pectoral region. Fluctuati m and translucency being very distinct, I introduced a trocar at the most dependent part in front, and drew off nearly a pint of pale, straw-colored and richly albuminous liquor. After closing the aperture with styptic colloid, and applying a cotton-wool compress, I requested to be informed of the progress of the case. I heard nothing of it for eight months. When the child was again brought to me last December, the tumor was larger than when first seen, and the contents, though still liquid, had undergone a very bloody change. The mass was no longer translucent, and the skin was uniformly bluish. I introduced two ordinary sized drainage tubes from back to front, at a distance of a couple of inches, and applied a tenax compress. A considerable quantity of reddish fluid oozed through the tubes, but as days elapsed the mass did not perceptibly lesson, and it became evident that something more must be deno to effect a radical cure. Dissection has proved that these congenital cystic growths in the neck are under the fascia; and in the particular case entire removal would only have been possible after a dissection attended with risk. With a view to effect a cure with the utmost safety, I removed the two small drainage tubes, and while my friend and colleague, Dr. Mackey, administered chloroform, I made an incision on the anterior aspect, a little below the middle line of the tumor, and pushed into its centre an india-rubber drainage tube, two inches long and a quarter of an inch in diameter; the anterior extremity of the tube projected slightly from the wound, and was kept in position by a loop of thread on each side secured by adhesive plaster. At the end of a week a great deal of irritation had been set up; the mass was hot and semi-solid; the child was feverish, and the discharge semi-purlent. The tube was now removed, and a linseed poultice applied. Within a week three separate collections of matter were evacuated by the aid of the lancet; fever subsided, a dry pad was applied with daily increasing pressure and the rapid decrease of the enlargement. No trace of it is now perceptible, and the child is perfectly well.—*S. Gamgee, F. R. S. E., Birmingham, Eng.*

*Coxalgia Successfully Treated by the Use of Sayre's Hip-Joint Splint—By. J. A. Draughan, M. D., Nashville, Tenn.—(Read before Nashville Medical Society, July 1st,* 1875.)—May 13, 1875. I was called to see a little girl about three years old, the mother having brought her to Nashville, Tenn., for medical advice. The parents had noticed that about three months previous she would become wearied on exercise, that there was considerable pain at the knee-joint, and that she would spend restless nights. As soon as I saw the case, and learned the history, I had no hesitation in pronouncing it disease of the hip-joint. The signs which presented were pain at the hip-joint, obliteration of the gluteo-femoral crease, slight flexion of the leg upon the thigh; the whole limp was somewhat abducted, and the semi-membranosus and semi-tendinosus muscles were contracted.

The first indication in the treatment was to use extension and counter-extension, which I did by means of the weight and pulley. This treatment I continued for three days and nights, until I was satisfied that the contracted muscles were relaxed, and that inflammation had, in a certain measure, subsided. I then removed the extension, and applied Dr. Lewis A. Sayre's "hip-joint splint."

The child, previous to the application of the splint, could not be moved in bed without producing excruciating pain. She was anæmic, and her nervous system very much shattered. As soon as I applied the instrument, and put on the necessary amount of extension and counter-extension with the apparatus, I stood the child on the floor; she was able to place the foot of the affected side to the floor, and bear the whole weight of the body on it without pain—a thing she had not done for four months. She was taken to the country, and a few days since her mother brought her down for me to examine, and to see that the instrument was properly adjusted, when I was amazed at the change which had taken place; the improvement was very marked. The child had been able to play in the open air; she no longer looked anæmic. The mother wrote me a few days after returning home that her child was improving rapidly.

The treatment of morbus coxalgia has, within the past few years, undergone a revolution. No longer are blisters, poultices and anodynes applied to the diseased joint, but the treatment is almost limited to mechanical measures, such as I have mentioned above.—*Virginia Medical Monthly.*

*Phosphorus in Neuralgia.*—Dr. Bradley (*London Lancet*) writes: "The remedy which has worked so speedy, and as it proved in the sequel, so permanent a cure, consisted of the so-called mother tincture of phosphorus, of which he was ordered to take five drops on the advent of an attack, and repeat them as occasion required.

"This tincture of phosphorus is a solution of phosphorus in ether, which dissolves about one per cent., so that each dose contained about one-twentieth of a grain of phosphorus—scarcely homœpathic according to old-fashioned notions; *mais cela va sans dire.*

"Not only was the pain relieved, but the frequency of the attacks was lessened, until from suffering a seizure two or three times a week, as he had for some years, he has now been entirely free for more than four months.

"Since the occurrence of this case, I have frequently employed this preparation of phosphorous, and have often found it of signal service in curing neuralgia; especially, it has appeared to me, in those subjects who add to a highly nervous temperament some cause of nervous waste; so that I have considered it probable that the neuralgia has, indeed, in these cases been, as Romberg styled it, "the cry of the hungry nerve for blood," or, rather, for its own special pabulum in the blood, and that the phosphorus has directly supplied this want."

*Surgery of Arteries.*—In a lecture delivered before the Medical Society of London, January, 1875, by C. F. Maunder, F. R. C. S., Surgeon to the London Hospital, and in which the lecture illustrated, and sustained his points, with a large number of cases. He adduces the following conclusions:

From the above cases of hæmorrhage we may conclude: 1. That no operation is to be performed when bleeding has ceased, unless a repetition of it would directly endanger life. 2. That the bleeding vessel is to be sought at the seat of injury, and to be secured, if divided, at both ends, either by a ligature or by torsion; if only wounded, by a ligature above and below the wound; or after section, by torsion. 3. That the injured vessel is only to be tied on the cardiac side of, and at a distance from, a wound in it, when the attempt to secure it at the wound has either been made and failed, or when such an attempt would be either anatomically injurious or pathologically useless. 4. That it is desirable to ligature the brachial artery, rather than both radial and ulnar, for secondary hæmorrhage from the hand. 5. That ligature of the brachial, while it stops bleeding, also arrests destructive inflammatory changes caused by useless local efforts to check hæmorrhage. 6. That blood flowing from the distal side of a wound in an artery, or ligature upon it, will in the lower extremity be often, in the upper extremity occasionally, venous in color. 7. That in malignant disease, when the growth cannot be removed and it is impossible to check bleeding by milder measures, the feeding artery may be ligatured in its continuity. 8. Where a part is more or less disorganised, and hæmorrhage renders repair very doubtful, amputation should be performed to arrest bleeding and remove a hurtful member. 9. Indirect compression will occasionally arrest severe bleeding. 10. That both the axillary and the femoral arteries may be wounded, and a pulse be felt at the extremity of the limb. 11. That a wound in an artery may be recognised by the warm blood impinging on the inserted finger. 12. That direct compression upon the bleeding point will often succeed *after* the main artery has been tied, though it failed *before;* and this fact is a justification for tying a main vessel.—*London Lancet.*

*Acidulated Gargles in Typhoid Fever.*—M. A. Netter, alluding to the buccal element in typhoid fever, and the beneficial influence of frequently repeated acidulated gargles, draws the following conclusions:

1. Call the attention of the patient to the bad odor of their mouth, and inform them that not only in it, but also in the nose, there is something being secreted which poisons the whole system.

2. Place at their disposition an unlimited quantity of a solution containing two hundred grammes of decoction of barley, thirty grammes of honey, and twenty-five grammes of vinegar. Let them gargle and rinse their mouth with this frequently, and also snuff it into both nostrils. When they have commenced with this, it will be found so agreeable that large quantities will be consumed.

3. The nurses should be instructed to encourage and assist the patients during this operation. Where the adynemia is very profound, the nurses should cleanse the mouth for the patients.—*Gaz. des Hop. and Trib. Med., No.* 308, 1874, *G. R. C.*

*Critique on the Esmarch Method.*—Mr. Chiene, in the *Edinburgh Medical Journal,* observes that the thoroughness of action of the elastic bandage is the chief objection to its use; the smaller arteries being so completely emptied that natural arrest of hemorrhage by the formation of external clot during the operation cannot take place. As a consequence, when the compressing agent is removed, many vessels bleed which would have been otherwise closed; and before the surgeon has time to secure them by ligature, much blood is lost, and the operation is unnecessarily prolonged. The complete anæmia of the limb takes away that guide which oozing of blood gives to the situation of the smaller arteries, which cannot, therefore, be tied until the circulation is restored. And when it is restored every small arte·y bleeds freely, and leads to further loss of blood. Mr. Chiene considers the plan of simply raising the limb to be preferred, because it is not so complete; a gentle oozing as a guide to the smaller arteries is not prevented, and enough blood is left to allow external clot to form. In cases where there are putrifaction-products in the limb, the compression of the bandage is hurtful, and here, again, raising the limb is to be preferred. He objects to the elastic tubing, that it does not allow of being relaxed and tightened at the pleasure of the operator, a serious obstacle in private practice, where the number of assistants may be limited; and he would rather use Petit's tourniquet than the band of Esmarch.—*Med. & Surg. Rep.*

*Removal of Foreign Bodies from the Throat by Injection.*—At a meeting of the Swedish Medical Society, December 22, 1874, Dr. Lamm showed a sharp-pointed, flat bone which he had removed from the throat of a man under circumstances which contradicted the use of forceps or the other instruments usually employed for this purpose. The method adopted was to pass a fine and very inflexible catheter to a point a little distance below the seat of pain. An india-rubber syringe was connected with the catheter, and warm water injected, at first slowly, and then with increasing force. The bone was suddenly expelled, and the water which returned with it was quite clear, showing that none of it had entered the stom·ch. It was supposed that the lower end of the œsophagus had closed spasmodically, and the bone was loosened and floated out by the distending force of the water pressing from below upwards.—*Medical Record.*

*A Pathognomonic Symptom of the Moribund Condition.*—Dr. John Shrady, in a paper upon the "Moribund Condition," recently read before the Yorkville Medical Association of this city, maintained that the earliest, and therefore most valuable symptom of approaching death, was the up and down movement of the trachea; that the inferior laryngeal nerve, owing to a partial paralysis, or impairment of its function, is concerned in the production of this phenomenon, and sounds the first note of alarm that the medulla oblongata is invaded.

This tracheal symptom is particularly prominent in fatal cases of uræmic convulsions, opium poisoning, apoplexy, and delirium tremens; the air then ceases to stimulate the glottis, the respiratory movements are impaired, and the lungs can no longer decarbonize the blood.

In pneumonia this symptom is of especial value, anticipating as it does alarming changes in pulse and temperature; while in phthisis, the doctor has known it to be a precursor of death three weeks in advance. Its presence or absence in membranous croup should be, inhis opinio n, an important element in the prognosis of a given case of tracheotomy.—*N. Y. Med. Jour.*

*Opium or Salicin in Diarrhœa* (*Detroit Review of Medicine and Pharmacy*, July, 1875.)—Dr. J. C. Bishop, after discussing the physiological action of opium and salicin, and giving a number of cases occurring in his own practice, comes to the following conclusions:

1st. Salicin is perfectly harmless, even when administered to very young children; opium is not so. 2d. Salicin increases the appetite and promotes digestion; opium destroys the former and retards the latter. 3d. Salicin may be administered to the most delicate stomach without any ill sequences, while opium is absolutely contra-indicated in many persons, who possess a peculiar susceptibility to its action. 4th. Salicin has no appreciable effect upon the brain, while opium induces a hyperæmia of that organ. 5th. Salicin possesses valuable antiseptic properties, while opium, if it possesses any, does so in a very feeble degree. 6th. Silicin is an antiperiodic, while opium has no notable effects in that direction. 7th. Silicin prevents the putrefactive changes in the contents of the bowel; opium does not.—*Medical Times.*

*A New Antiseptic Dressing.*—The *Edinburg Medical Journal*, of September, contains a formula for a new antiseptic dressing employed by Lister in a case of rodent ulcer of the face: Boracic acid in fine powder, one part; white wax, one part; paraffin, two parts; almond oil, two parts. The ingredients, after being mixed by melting the wax and paraffin, are stirred in a warm mortar till the mass thickens, and then set aside to cool, after which the fine substance is reduced in a cold mortar, in successive portions, to a uniform soft ointment. This is spread thin on a fine rag, and when the almond oil leaves it, as it soon does through the capillary attraction of the porous external dressing, a smooth, firm layer remains, which can be separated from the skin without leaving any greasy substance adhering, and does not at all confine the discharge which, while freely shed, is perpetually supplied with a sufficient quantity of boracic acid to insure absence of putrefaction, while not preventing cicatrization.

Another, and, it is thought by Dr. Henry (*London Med. Record*,) a better application in cases of this nature, is an ointment composed like the one above described; except that instead of one part of boracic acid, it contains half the quantity of salicylic acid, the antiseptic virtues of which have been recently discussed by Prof. Kolbe, of Leipsic. Salicylic acid, while possessing very remarkable antiseptic powers, is even less irritating than boracic acid.

*Ovulation Without Menstruation.*—At a late meeting of the Obstetrical Society of Edinburgh, Dr. James Young mentioned the following interesting case as an example of ovulation without menstruation: The lady referred to was married on the 13th of June, 1867, at the age of 25 years. She menstruated on the 13th of July, and her first child was born on the 4th of April, 1868. The patient stated that she nursed the child thirteen months, and then menstruated six times—till November, 1869. The second child was born on the 15th of August, 1870, was nursed five months, when the baby died. From November, 1869, until January, 1875, the patient had never menstruated; during that period a third child was born, 31st October, 1871, while a fourth child was born on the 12th September, 1873, and a fifth pregnancy was then going on, each child having been nursed twelve months.—*Obstetrical Journal, April*, 1875.

*Browbeating Hysteria.*—A correspondent of the *Boston Medical and Surgical Journal* gives the following account of the treatment of a typical case of hysteria by Dr. Weir Mitchell:

Patient was a young lady who came to the doctor from Rhode Island for treatment. She had been in bed for months. The medical experience of Rhode Island had been exhausted. Dr. W. A. Hammond advised a longer continuance in bed. Dr. Mitchell made three visits ere he began treatment. The peculiarities of the case were spinal weakness and an inability to straighten the lower extremities. At his fourth visit the doctor requested his patient to straighten her limbs. "But I can't." "But you *can*. Are they never straightened at night?" "Yes, doctor. No one ever asked me that question." The legs were straightened with but little difficulty. "Now be kind enough to sit up." "But that is impossible; I have not been able to do it for two years." "You are able now. Please sit up." Patient sat up. "Bring her wrapper, hose and slippers and put them on; put on a necktie; belt her waist. Now I wish you to stand." The patient now began to cry. "Good morning," said the doctor, taking his hat. "Where are you going, Doctor?" "I am going away. I never attend patients who do not obey me." "Come back, Doctor; I will obey you." "Then please stand up." She stood up. "But, Doctor, it makes me so dizzy." "I expected it. Take my arm." She took his arm. He led her slowly out of the room, down stairs and out of doors. She returned without aid, and *did not go to bed again.* She was cured. This is given as a sample of the doctor's treatment of hysteria. He is never unkind, never rough, but inflexible, quick in manner, decided in speech, yet gentle and exceedingly polite.—*Baltimore Physician and Surgeon.*

*Enemata of Bromide of Potassium in Obstinate Vomiting.*—Dr. Girabetti (*Medical News and Library,* Auguat 8, 1874,) has obtained the very best results from the administration of enemata of bromide of potassium, in doses of from one-half to two drachms, in cases of obstinate vomiting attending the pregnant state. The same drug, also administered in enemata, has been very successful in the hands of Dr. Laborde, of Paris, in obstinate vomiting connected with the disease of the stomach, liver and intestines.—*London Lancet.*

*Ergot as an Anti-galactagogue.*—A Russian physician, Dr. Schtscherbinenkoff, has made the interesting observation (*Centralblatt fur Chirurgie*, May 8, 1875) during an epidemic of secale cornutum in the district of Simbirski, that amongst the symptoms of ergot-poisoning is the diminution or complete arrest of the secretion of milk in lactating women. The same result was found to occur in cows that had been fed on meal which contained ergot, or that had been littered with carelessly threshed straw which still contained some affected ears. Schtscherbinenkoff conceived the idea of employing it as a remedy in cases of threatened abscess of the breast, and carried it out in many cases with great advantage. Two multiparæ who had suffered at each previous confinement from abscess of the breast, took some of the drug with the happiest result, as soon as swelling began to appear, accompanied by congestion of the gland with milk. He has also administered ergot and quinine, in doses of three grammes of each, twice or thrice a day, in cases of so-called milk-fever, and has found the drug useful in cases of swelling of the breast accompanied by fever, in other than puerperal women, as well as in those where it is required to wean the child either at the normal or at an earlier period. He has exhibited as much as a drachm of ergot daily, for a week, without observing any unpleasant result. *Med. & Surg. Rep.*

*Eucalyptus and Tobacco.*—A Captain in the French army, according to *Science Pour Tous*, not being able to abandon the use of tobacco for smoking, although its use caused him serious inconvenience, has for several years mixed a few dried leaves of *eucalyptus globulus* with the portion or the weed which he is in the habit of using daily. Since he has employed this mixture, he asserts that he has experienced neither vertigo, headache, nor pain in the stomach. He recommends this means for preventing the disagreeable effects of the pipe, without compelling the individual to renounce his old habit.—*Medical and Surgical Reporter.*

It is stated that nearly all the men selected for the British Arctic Expedition are of a fair complexion. Scoresby's test was an ingenious and probably a good one. Each candidate was obliged to stand with his bare feet upon a cube of ice, and those who endured longest were chosen.—*Med. Times.*

*Administration of Purgatives by the Hyperdermic Method.*—Prof. Luton, of Rheims, (*Mouvement Medical,*) for the purpose of relieving himself of an attack of hemicrania, resorted to the hypodermic use of muriate of morphine, of which he had previously neutralized an acid solution with calcined magnesia, to prevent subcutaneous irritation ; of this he injected a syringeful. Having before had a costive tendency, he was now attacked by a mild diarrhœa. He then instituted further experiments on himself with an injection of sulphate of magnesia, (1:10,) which resulted in diarrhoea. In another man, with constipation, in whom aloes and rhubarb were inert, he injected 0.1 gramme of sulphate of magnesia in 1 gramme of water, after which the patient had two stools. Injections were also made with the aqueous extract of aloes, (1:10,) and this seems to be borne better by the subcutaneous tissue than the former remedy. The experiments of Vulpian and Corville on dogs showed that the animals after this treatment were attacked with intestinal catarrh. In four patients suffering from vomiting and constipation, injections of sulphate of magnesia caused looseness of the bowels and cessation of the vomiting. From this Prof. Luton concludes that these injections paralyse the antiperistaltic and stimulate the peristaltic movements, and he suggests their use in the vomiting of pregnancy, seasickness, etc.—*N. Y. Med. Jour.*

*Charcot on the Relief of Hysterical Seizures by Compression of the Ovaries.*—According to Charcot, most hysterical seizures are preceded by an aura starting from one or both of the ovaries, and he finds that pressure on the organ indicated causes immediate arrest of the seizure. He illustrated this action upon a patient in the Salpetriére affected with hystero-epilepsy. The seizure recurred, however, the moment the pressure was taken off. In order to keep up the pressure for a longer time than would be possible with the unaided hands, he recommends an apparatus like a tourniquet. The pressure is to be made in the situation and direction requisite in compressing the iliac artery, and, in fact, this vessel will be felt pulsating under the finger.—*Gaz. Med. de Paris. Nov.* 5, 1875.

*Solution of Camphor in Erysipelas.*—In the *Gazetta Medica de Bahia* it is said that a few drops of a solution of equal parts of gum camphor and ether, applied from time to time to an erysipelatous surface will, in the majority of cases, effect a cure.

*Prevention of the formation of Milk.*—Dr. Peaslee said that he had received from Dr. W. R. Wilson, U. S. Army, stationed at Fort Wayne, a communication in regard to the treatment of the breasts in cases where it was desirable to prevent lactation. Dr. Wilson wishes him to get the opinion of the Academy on the subject. Dr. Wilson's method consisted in strapping the breasts tightly after delivery, by means of strips of adhesive plaster. Dr. Peaslee said since his attention had been directed to the matter by Dr. Wilson, he had tried it in five cases of stillbirths, where it was desirable to suppress the milk. In all of the five cases the results were perfect. The method of using extract of belladonna had never been satisfactory, and suggestion of Dr. Wilson was very important.—*N. Y. Med. Jour.*

*A Remarkable Instance of Scarlatina lContagion.*—On the 10th of June a dinner party and reception were given at a fashionable house at South Kensington, in London. No illness of any kind had been in the house previously. Nevertheless, within two or three days afterwards, out of sixteen persons who were present at the dinner, six were attacked with scarlatina, and several of those who were present at the reception only were attacked with the characteristic sore-throat. Two of the servants were attacked. There is a *suspicion* that the cream furnished on the occasion may have been the conveyer of the contagion, but the investigation, which at last accounts was still going on, had not settled upon the source from which the poison came, or the vehicle of contagion.—*N. Y. Medical Record.*

*Local Use of Tannin.*—Dr. Miall (*British Medical Journal*, November 7, 1874,) gives the following facts from his experienc in the local use of tannin: He uses a concentrated solution. It is prepared by adding to one ounce of tannin six drachms of water. The solution is a thick fluid, that keeps well. It is used to convert living skin into leather. For wounds it is a far superior dressing to collodion. If applied by a brush and allowed to dry, it soon forms a pellicle, which excludes the air and gives ease to pain. It may be applied to ulcers, cracked niples, enlarged tonsils, bleeding warts, chronic phlegmonons, erysipelas, polypus of ear, with excellent results.

*Salicylic Acid in Diptheria.*—Dr. Fontheim has treated 31 patients of diptheria with salicylic acid. The severest cases were cured after eight days' treatment; the milder ones after 2, 3, and 4 days. Of the 31 cases, none died. There occurred no cases of diptheritic inflammation of the kidneys, nor were there any cases of paralysis of the palate. In the severe cases, Dr. F. ordered the affected parts to be rubbed, every three hours, with a sponge, dipped in a solution of salicylic acid, and to take as regularly, a teaspoonful of the same solution. The formula used was ℞. Salicylic acid, 2 grammes [about ʒ ss], Fountain water 200. grammes [about ℥ vij], Alcohol, q. s. [to make f℥vij]. Salicylic acid passes rapidly into the urine, and gives, with chloride of iron, a blue or violet reaction.—*Va. Med. Monthly.*

*A Male Girl.*—An individual about 20 years of age, who had always been considered and treated as a girl, stated that for the last two years she (or he) had known his (or her) true sex. Now, in order to marry, a change was necessary. An examination proved him to be a male in every particular, and the following condition of the sexual apparatus was observed: The scrotum was drawn in the median line; it contained testicles, and on the right side an inguinal hernia. In the erect posture and with closed thighs the penis could not be seen, but when the thighs were separated, it was found imbedded in the raphe of the scrotum, and its direction backward and downward. The glans was perfectly free, and the individual stated that he had already accomplished the act of coition very satisfactorily on two occasions.—*Allg. Wiener Medizinische Zeitung, June* 1, 1875.

*Acute Hematuria Treated Successfully by Local Applications.*—Dr. E. L. Keyes (*Archiv Dermatology,* October, 1874,) describes a case of unusual interest: A young, robust man was attacked by hematuria. After a long course of general treatment, without avail, a careful examination was made, proving that the source of the blood was the prostatic sinus.

This sinus was cauterized by the application of solid nitrate of silver. In two days the application was renewed. The blood stopped in twenty-four hours. Symptoms of crystitis of the neck came on, but gradually disappeared, and the patient did not again pass blood with his urine.

*Phosphorus as a Stimulant.*—Dr John Brunton, of London, gave phosphorus to a patient under the following circumstances: "On examination I found the characteristic rose-colored spots of typhoid fever, and he had all the other symptoms present. His fever continued gradually to increase, when he said to me, 'I am going to die,' and he looked like it. His conjunctivæ were injected, his breath cold, his skin cold and clammy; pulse 48, very weak and compressible; voice whispering; temperature 06.4°. He was in a condition of extreme depression. I at once administered phosphorus, in doses of one twelfth of a grain, every two hours, and I was surprised to find on my next visit, eighteen hours after, when he had taken three quarters of a grain of the drug, that he had quite revived. His skin was comfortably warm, eyes not so suffused, voice more natural; pulse 72; temperature 99°. I immedimediately stopped the phosphorus and gave nitric acid. Since then he has gone on prosperously, and is now convalescent. Heroic doses need careful watching, and I am sure the formula appended is most stable and active, and it is not unpalatable. I do not think I should care to go beyond one or at most two grains of the drug, divided over two days. ℞. Etheral tinct. phosp. (gr. ⅓ to ʒj), ʒiij.; spt. vin. rect., ℥ss.; glycerin. anhydr., ad ℥iss. One teaspoonful as a dose."—*American Practitioner.*

*Iodine as a Substitute for Iodide of Potassium.*—It is found that in some cases, where iodide of potassium is not tolerated by the stomach, iodine itself can be administered in the form of pills.

The iodine is first suspended in albumen, and afterward the combination is dried and powdered. In this way one-quarter grain of iodine can be made into a pill, and two and a half grains can be given daily, without disordering the stomach. This method was obtained from Paris, and proves of much service in relieving tertiary symtoms.—*N. Y. Medical Journal.*

*To Prevent the Marks of Small Pox.*—Touch the pustules with glycerin and then sprinkle with a powder of red oxyd of mercury one part, sublimed sulpher four parts. An Italian physician says this will cause the pustules to dry and desquamate in a few days, without leaving the slightest mark.—*Pacific Med. & Surg. Journal.*

*Treatment of Acute and Chronic Bronchitis and Asthma.*—Dr. Spurgin, of York, states that he has tried iodide of potassium in over a hundred cases with almost invariable success; in fact, with such success that patients have expressed themselves by saying, "It has acted like a charm." Others have said that no medicine ever had any real effect upon their complaint before. Iodide of potassium has a marked effect upon the breathing, reducing the frequency of the respirations; perhaps also, he thinks, overcoming spasms. Almost after the first dose patients have stated they have felt the medicine touch their complaint. Dr. Spurgin usually prescribes it with carbonate of ammonia, and when the cough is very troublesome, adds tincture of belladonna and ipecacuanha wine. In one very bad case of broncho-pneumonia he tried iodide of potassium with tincture of hyoscyamus and ammonia, and the respirations were quickly and astonishingly reduced from forty in a minute to less than half that number. He adds that he has purposely given a mixture containing ammonia, belladonna, ipecacuanha wine, spirit of sulphuric ether, etc., without iodide of potassium, and has not found much benefit result from their use. After this he has added the iodide of potassium, and found the patient relieved almost at once.—*British Medical Journal.*

*Delirium in Typhoid Fever.*—In the treatment of this complication, remember that it is owing to brain-irritation, not to inflammation. There is one great remedy for this, and it is alcohol; alcohol is the remedy for nervous irritation in typhoid fever, and, in fact, in any fever. How it acts is not certainly known, but it may be laid down that it is an important point in the treatment of all fevers. It allays nervous irritation and soothes the nervous system.—*Sir W. Gull.*

*Warts.*—Dr. Guttceit recommends rubbing warts night and morning with a moistened piece of muriate of ammonia. They soften and dwindle away, leaving no such white mark as follows their dispersion with lunar caustic.—*Eclectic Medical Journal.*

*British Medical Association.*---The next meeting of the British Medical Association will be held at Brighton, under the presidency of Sir John Cordy Burrows.---*N. Y. Med. Rec.*

*Absolute repose in Tetanus.*—Prof. E. de Renzi draws the following conclusions from his observations of tetanus:

1. Light renders the tetanic contractions of animals and man more frequent and intense.

2. It can be demonstrated experimentally in animals that absolute repose, during the absence of all stimulus, retards the tetanus and renders it less fatal.

3. Of three cases of severe tetanus, treated almost exclusively by absolute repose, two cases were cured. The patients were kept isolated in a dark room; all noise or other stimulus or irritation was avoided, except such as was caused by the administration of of food and beverage at long intervals.

4. In one case death resulted, notwithstanding the administration of large doses of hydrate of chloral and several hypodermic injections of woorara. It would appear that the chloral increases the difficulty of respiration, which is already affected by the disease.

5. In the actual condition of science, absolute repose shows itself to be the principal remedy in the treatment of tetanus. The removal of stimulus should, however, be as complete as possible, and be recognized as an important accessory.

6. In two cases of traumatic tetanus, the first severe tetanic manifestations occurred a few hours after the amputation of the injured limb. This shows that amputation, instead of impeding the further development of the disease, renders it more intense from the irritation of the nerves of the amputated part.—*Gaz. Med. Ital. Lomb.*

Dr. Van Bibber, of Baltimore, reports the case of a gentleman who was in the habit of taking, for eight or ten days consecutively, not less than sixty grains of morphia subcutaneously, together with three pints of whisky and eight or ten cigars---*Clinic.*

*Insecticides.*---A tincture of nux vomica prepared with caustic solution of ammonia is recommended as the best of insecticides. Bed-bugs, cockroaches, etc., are at once destroyed by it, and it is said that if a horse's harness be painted with it, the flies will avoid him. We have found carbolic acid effectual. Against ants, castor-oil is found an entire protection.---*Exchange.*

*Use of Quinine in the Treatment of Infantile Diseases.*—In a paper in the *Deutsche Klinik*, 1874, Dr. Rapmund contends that quinine and cold affusions are the most energetic and most certain antipyretics. He gave quinine in four cases of scarlet fever, eleven cases of measles, two cases of small pox, three cases of erysipelas, nine cases of lobular pneumonia, and three follicular interitis. When a sufficient dose had been administered the temperature and frequency of the pulse fell, and a calm, prolonged sleep ensued. Quinine has great power in rendering the course of febrile diseases benign. In the treatment of erratic erysipelas by quinine, Rapmund has been as successful as Vogel. In the treatment of lobular pneumonia of infants by quinine, the author obtained seven cures out of nine cases. It is in the stage when the febrile simptoms are acute, and the temperature and pulse-rate higher than normal, that quinine is indicated. In hooping-cough, quinine diminishes the violence of the attack and promotes rest at night. It also appears to prevent complication, and to render the course of the disease benign.—*A Practitioner.*

*Xanthium Strumarium.* (*The American Medical Journal*, July, 1875).---Dr. William Fulton reports the xanthium strumarium, or common cockle-bur, as one of the most reliable of diuretics. He has found it a specific in all cases of urinary difficulties in pregnancy, and prescribes it in all cases where there is scalding in urinating. He uses it in the form of a tea, or as a saturated tincture, of which he adds about two drachms to a tumbler of water, and gives one tablespoonful every two or three hours.---*Phil. Med. Times.*

*Cough and Sweating in Phthisis.*---Dr. Little, of Dublin, recommends the following combination for the relief of the distressing cough of phthisis, and for the diminishing the sweating: Acetate of morphia, 2 grains; liquor of atropia, 6 minims; dilute hydrocyanic acid, 36 minims; syrup of Virginia Prune to an ounce and a half. A measured drachm to be taken, unmixed with water, on going to bed, and once again during the night if necessary.---*Canada Lancet.*

THE press: in all civilized communities the alma mother of charlatanism.—*Gazette Hebdomadaire.*

*Salicylic Acid.*---The *Boston Medical and Surgical Journal*, March 11, contains an abstract of a letter received by Prof. Horsford from Kolbe, of Leipsic, in which the latter gives various details of interest relative to the use of this product. In the lying-in hospital of Leipsic it seems to have superseded carbolic acid as a disinfectant for the hands, vaginal douching, application to ulcera puerperalia, etc. It is used in solution in water of one part in three hundred to one part in nine hundred, or as a powder mixed with starch in proportion of one part in five. Kolbe recommends the trial of this remedy internally in the various zymotic diseases, in pyæmia, bites of dogs, etc. In order to prove its inocuosness, he administered to himself and a number of students various doses of the acid, up to twenty-three grains a day. The following is recommended by Wunderlich as a convenient formula for its administration: ℞. Acidi salicylici, gr. xvi.; Ol. amygdalæ dulc, f℥ vi.; Acaciæ gummi, ℥ iii.; Syrupi amygdalæ, f℥ viiss.; Aq. aurantii flor., ad f℥ iv. M. Salicylic acid is very little, if at all, absorbed through the skin.---*Phil. Med. Times.*

*Longevity in England.*—The mortality returns for England for the year 1872 show that 195 men and 433 women died at 95 years old and upwards. Of this number 24 men reached or exceeded 100 years, one attaining 111 years. Of the women, 51 reached or exceeded 100 years, two having died at the age of 107. Of the whole number remaining, men and women, two died at 101, two at 102, one at 203, two at 104, and one at 105.—*N. Y. Medical Record.*

*Solution for Hypodermic Injection.*—If to each ounce of magendie's solution of sulphate of morphia five grains of hydrate of chloral is added, the solution will keep without deterioration in strength or quality for an indefinite length of time. The addition of the chloral prevents fermentative change, and does away with the necessity of making a fresh solution every few days. T. C. S.

THE following combination was used extensively by me in 1866 in the treatment of cholera at this place. I also lent the prescription to physicians and friends in the interior, who found it to act well in cholera, cholera morbus, diarrhœa, etc. ℞. Creasote, gtt. viij.; ol. minth pif. gtt., viij.; carb. soda ʒj.; aq. gs. ad. ft, ℥iv. M. Sig. one tablespoonful for adult in urgent cases every half hour.

*The First Authentic Satire on Physicians.*—Dr. Dugan Clark, in an address to the graduating class of the Indiana Medical College, gives the following as the first recorded joke perpetrated on that much be-joked class—doctors. It is too good to be lost. He says:

H. B. L.

"And while speaking of Biblical medicine, I may remark that those persons who, *when in health*, are very much in the habit of making satirical speeches about the doctors—using their wit to show, in many ways, how intimate the connection is between the calling of the physician and that of the undertaker—will find themselves anticipated and excelled by the author of the Book of Chronicles.

"I would not for a moment insinuate that the sacred writer was intending to perpetrate a joke at the expense of our profession, but certainly the following passage sounds wonderfully like it: 'And Asa, in the thirty and ninth year of his reign, was diseased in his eet, until his disease was exceeding great; yet in his disease he sought not to the Lord but to physicians. *And Asa slept with his fathers.*'"

*Boston Medical Library.*---The phyisicians of Boston held a meeting on the 20th of August for the purpose of organizing a medical library, to consist of medical and scientific books, journals, pamphlets, for ready reference. Offers have been made of pamphlets and journals, and of one or two private libraries. More than one hundred members have already joined, and the rooms are to be opened in October..---*N. Y. Med. Rec.*

DR. BRAND, a physician of St. Catharines, sixty miles above Memphis, Tenn., was lately shot in the back of his head and instantly killed, by an unknown assassin, while sitting in his office. *N. Y. Medical Record.*

A VERITABLE Cyclops is reported to be in London. His only eye is in the middle of his forehead. His name is Piper Wilson, aged twenty-two, and he came from Australia.

A SCHOOL for nurses is to be opened at Charity Hospital, New York. The course is of Two years' duration.—*Medical Times.*

*A Certain Sign of Death.*—The most certain sign by which apparent death may be distinguished from real death, is held by Monteverdi to be the wine-red color of the skin, which is produced by a hypodermic injection of ammonia into the body. The theory on which this fact is founded is that the last act of human organic life consists in absorption. This function does not cease until after complete cessation of the capillary circulation. Liquor ammoniæ, is the only reagent yet known which is reliable as an indication of the activity or cessation of absorption, and, therefore, of the capillary circulation. The brightness and size of the resulting spot is a measure of the vitality which still exists. In a dead body the injection does not produce any reddish discoloration of the skin whatever.—*Giorn. Ven. di Sci. Med.*

*Goa Powder in Skin Diseases.*—Dr. Blanc has treated tinea circinata with success by the external administration of goa powder, which destroys the tricophyton with great rapidity. This goa powder, the nature of which is not as yet thoroughly understood, is constituted in great part of chrysophanic acid.

It is employed by simply sprinkling it night and morning over the affected parts, and a cure is obtained by this means in from four to five days. Dr. B. proposes the use of this powder in cases of tinea tonsurans.—*Journal de Therap.*

*Sterility* (*The Doctor, July* 1, 1875.)—Among the causes of human sterility, Dr. Sims mentions the projection of the neck of the uterus into the vagina. If the neck, says he, projects half an inch, sterility is probable; if one inch, it is almost certain; if one inch and a half, it is inevitable. Dr. Giuseppe, of Turin, maintains that in two women out of three the neck of the uterus projects naturally half an inch. The subject is one of importance, and the disparity of opinion merits careful consideration.—*Medical Times.*

*Bullet Wound of the Stomach and Kidney* (*Boston Medical and Surgical Journal,* August 5, 1875.)—Dr. M. E. Jones reports a case of bullet wound of the stomach and kidnev followed by hæmaturia. Morphia and gallic acid were employed, and the patient made a good recovery.

